# The Future of PET Imaging in Multiple Sclerosis: Characterisation of Individual White Matter Lesions

**DOI:** 10.3390/jcm14134439

**Published:** 2025-06-23

**Authors:** Chris W. J. van der Weijden, Jan F. Meilof, Anouk van der Hoorn, Erik F. J. de Vries, Wia Baron

**Affiliations:** 1 Department of Radiology, University Medical Center Groningen, University of Groningen, 9713 GZ Groningen, The Netherlands; a.van.der.hoorn@umcg.nl; 2 Department of Nuclear Medicine and Molecular Imaging, University Medical Center Groningen, University of Groningen, 9713 GZ Groningen, The Netherlands; e.f.j.de.vries@umcg.nl; 3 Department of Neurology, Martini Hospital, 9728 NT Groningen, The Netherlands; j.f.meilof@umcg.nl; 4 Department of Biomedical Sciences, Section Molecular Neurobiology, University Medical Center Groningen, University of Groningen, 9713 AV Groningen, The Netherlands; 5 MS Centrum Noord Nederland, The Netherlands

**Keywords:** multiple sclerosis, neuroprotection, oligodendrocyte progenitor cell, positron emission tomography, radiotracer, remyelination

## Abstract

Multiple sclerosis (MS) is a multifaceted inflammatory, demyelinating, and neurodegenerative disease typified by lesions with distinct hallmarks in the central nervous system. Dysregulation of micro-environmental factors, including extracellular matrix (ECM) remodelling and glial cell activation, has a decisive effect on lesion development and disease progression. Understanding the biological and pathological features of lesions would aid in prognosis and personalised treatment decision making. Positron emission tomography (PET) is an imaging technique that uses radio-labelled tracers to detect specific biological phenomena. Recent PET hardware developments enable high-resolution, quantitative imaging, which may allow biological characterisation of relatively small MS lesions. PET may complement MRI by offering objective, quantitative insights into lesion characteristics, including myelin density, inflammation and axonal integrity. Moreover, PET may provide information on lesion traits supporting decision making on upcoming therapeutic strategies for progressive MS, such as the availability of oligodendrocyte progenitor cells and ECM composition that affect remyelination and/or axon regeneration. This review explores the cellular and molecular ECM signatures and neuropathological processes of white matter MS lesions, discusses current and potential novel PET targets that may help characterise MS lesions in vivo, and addresses the potential of PET as a decision tool for selection and evaluation of therapeutic strategies, with a focus on remyelination.

## 1. Introduction

Multiple sclerosis (MS) is the most common neurodegenerative disease among young adults [1] and is characterised by chronic inflammatory demyelinated lesions in grey and white matter [2]. Given the heterogeneous distribution of lesions within the central nervous system (CNS), MS is associated with a broad spectrum of symptoms. The most typical symptoms are those involving the optic nerve, the urinary tract, and those resulting in sensory and motor deficits [3,4]. MS treatments are generally focussed on suppression of the peripheral inflammatory response, which adequately limits infiltration of peripheral immune cells and consequently inflammation-mediated demyelination, but neglects neuroprotection and lesion repair, including regeneration of myelin and axons [3,4]. Currently, several remyelination-promoting agents are being tested in clinical trials [5,6], but it remains difficult to prove their effectiveness, due to the lack of knowledge of the dependence of the type of MS lesion on disease progression and the lack of adequate imaging methods to visualise remyelination.

Histopathological analysis of MS lesions reveals different lesion types within and between grey and white matter, which may affect diagnosis and therapy selection. With routine MRI gadolinium-enhanced T_1_-weighted (T_1_w), only a gross distinction can be made between lesions, being either inflammatory active or inactive [7]. MRI-based lesion activity primarily describes blood–brain barrier (BBB) disruptions caused by acute inflammatory processes. These processes involve antigen-presenting cell-mediated activation of CD4-positive T cells, followed by activation of B cells, CD8-positive T cells, and macrophages [8], and result in immune cell and antibody-mediated injury of myelin sheaths. Although the identification of the inflammatory activity status of MS lesions is a huge step forward in diagnosis and characterisation of MS, the accuracy of histopathological assessment has still not been met. More knowledge of the cellular and molecular composition of individual lesions is necessary to adequately assess lesion prognosis, select effective treatment strategies and improve treatment efficacy monitoring. For example, a necessary step for efficient remyelination is the recruitment of activated oligodendrocyte progenitor cells (OPCs) to demyelinated lesions, followed by their maturation into myelinating oligodendrocytes [9,10]. Post-mortem histopathological analysis reveals that OPCs are not present in all MS lesions [11,12,13,14]. Although surviving pre-existing mature oligodendrocytes can also contribute to remyelination [15,16], they are less effective than newly maturated ones [17]. This indicates that different therapeutic remyelination strategies may need to be considered. Moreover, OPCs that are present in MS lesions face a non-permissive micro-environment, which makes them appear quiescent [10,13,18,19]. Micro-environmental aspects in lesions seem to play a major role in MS pathogenesis. For example, disturbances in the balance between extracellular matrix (ECM) proteins and matrix metalloproteinases (MMP) can either promote demyelination and axonal degeneration or promote remyelination and enhance axonal survival [20,21,22]. Thus, the cellular composition and molecular factors in MS lesions are important for a thorough characterisation of MS lesions and selection of treatment strategies; assessing these parameters is yet beyond the capabilities of MRI.

Characterisation and identification of cellular and molecular processes underlying MS lesion pathology in living patients may be possible with PET imaging. PET uses a radio-labelled molecule (PET tracer) that is able to bind to biomarkers that are specific for the biological process of interest, which enables imaging and quantification of the biological target [23,24]. The high sensitivity of PET makes it exquisitely suitable for in vivo lesion characterisation. Information about lesion characteristics, in combination with radiological and clinical evaluation, could further support decision making on treatment strategies and be used to evaluate treatment responses. In this narrative review [25], we first provide an overview of the cellular composition and molecular extracellular matrix (ECM) environment that can be used to classify MS lesions. Next, we outline white matter lesion determinants that are important for disease prognosis and treatment options, focusing on strategies to promote remyelination. Finally, potential applications of PET imaging to characterise the dynamic cellular and molecular alterations in white matter lesions are discussed. Patient stratification based on PET imaging of individual white matter lesion characteristics may be used in the future to tailor therapy decision making in trials and in clinical practice.

### Search Strategy

A comprehensive PubMed literature search was conducted, focussing on (1) identifying key cellular and molecular aspects of MS lesions, including inflammation, remyelination failure, neurodegeneration and ECM, (2) the use of PET imaging with emphasis on studies contributing to MS lesion classification, and (3) novel PET tracer delivery strategies to the CNS. The focus is on white matter MS lesion characterisation, since literature on environmental and biological aspects of the different grey matter MS lesions is still scarce. Additional relevant articles were identified through reference checking. While priority was given to the most recent literature to capture current advances, older, foundational studies were not excluded when relevant. An iterative approach was used, involving continuous evaluation and refinement of search results to ensure relevance and comprehensive coverage.

## 2. Multiple Sclerosis: Heterogeneity in Clinical Course and Pathology

### 2.1. Clinical Heterogeneity

MS is a complex chronic disease, encompassing inflammatory, myelin, and neurodegenerative processes. These pathophysiological processes are involved in episodes with a temporary increase in neurological symptoms, so-called relapses, and in disease progression [4,26]. In MS, there are two distinct disease courses considered. Relapsing remitting MS (RRMS) is the most prevalent form, with an 85% occurrence, that usually starts in young adults. RRMS is typified by active lesions, which are identified as gadolinium enhancement on T_1_w MRI due to disruption of the BBB, resulting in gadolinium accumulation within the tissue [7,27]. This perturbation of the BBB is deemed to result from inflammatory processes [28,29]. After ~3 weeks, the lesion no longer shows gadolinium enhancement, likely because BBB function is restored, and the lesion is then classified as MRI-inactive [30]. Clinical symptoms (such as paraesthesia, bladder dysfunction, bowel dysfunction, dysarthria, ataxia, tremor, optic neuritis, fatigue) emerge during relapses, but subsequently diminish over time during remission [4,29]. A recent addition to the definitions of the clinical course of MS is the concept of active versus not active disease based on the presence of relapses or MRI activity. Thus, patients can be labelled active RRMS or inactive RRMS. When relapses and remissions no longer occur and axonal damage increases gradually, MS patients are considered to have entered the secondary progressive (SP) stage of MS. Ten to 20 years after clinical onset, around 50% of the RRMS patients have converted to SPMS [31,32]. A minority of patients present with primary progressive MS (PPMS), which has a later onset than RRMS. In PPMS patients, clinical symptoms are slowly progressive from disease onset without relapses [29]. Neurodegeneration is more dominant in progressive MS, and the effect of peripheral inflammation is less prevalent [28]. Patients with progressive disease are further classified based on the presence of recent documented increases in disability. Thus, patients can be labelled as having active or inactive progressive MS, with or without progression. These subclassifications can be added to the clinical data when studying the relevance of specific MS lesion types. More recently, several studies analysing large groups of well-documented patients with MS observed a more insidious increase in disability even in people with RRMS without clinical or radiological signs of disease activity. This progression of disability has been termed silent progression [33] or progression independent of relapse activity (PIRA) [34]. These clinical observations suggest that in (some) patients with RRMS, neurodegeneration or low-grade inflammation is already present early in the disease.

Current MS treatments aim to reduce peripheral inflammatory activity to limit immune cell infiltration into the CNS and consequently demyelination. These treatments do not halt disease progression and are therefore only effective in RRMS. Consequently, therapies aimed at neuroprotection, such as overcoming remyelination failure and axon regeneration, are needed. Remyelination can be executed by both newly formed and pre-existing oligodendrocytes [10,15,16] and prevents axon (secondary) degeneration and restores saltatory nerve impulse conduction. Notably, in experimental models, and likely also in MS lesions, the remyelinating capabilities of pre-existing surviving mature oligodendrocytes are less efficient than the remyelination potential of mature oligodendrocytes newly formed from OPCs [16,17]. When axonal integrity is lost, as evident in advanced MS, axon regeneration is required to resolve functional deficits. It is currently not possible to predict which patients are at risk for a progressive and more severe course of the disease, and which therapeutic approach should be used to promote remyelination. Disease severity and remyelination failure are linked to the specific pathogenesis of MS lesions [35,36], and a better characterisation of MS lesions may enable prognosis, as well as better assessment of the kind of neuroprotective therapy to be considered.

### 2.2. Distinct Cellular Hallmarks of Demyelinated White Matter MS Lesions

**Figure 1:**
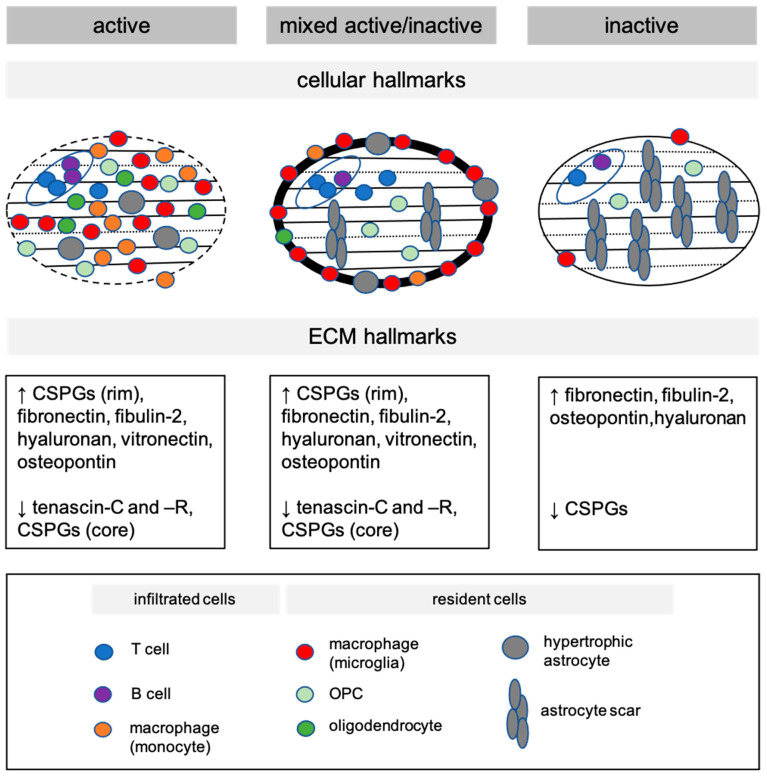
White matter multiple sclerosis lesion characterisation based on cellular composition and extracellular matrix (ECM) hallmarks. The ‘open’ red oval depicts a blood vessel with perivascular space, in which T and B cells accumulate. Within the parenchyma of active lesions, T cells, peripheral monocytes and resident microglia-derived macrophages, as well as hypertrophic astrocytes, oligodendrocyte progenitor cells (OPCs), and mature oligodendrocytes are present. Mixed active/inactive lesions have a rim, which predominantly consists of microglia, and a hypocellular lesion core consisting of an astroglial scar, OPCs and T cells. Inactive lesions contain predominantly astroglial scars, lack of monocytes, and low microglia numbers. Arrows pointing upward indicate an increase, while arrows pointing downward indicate a decrease. Note the distinct ECM composition between and within the different white matter lesions CSPGs—chondroitin sulphate proteoglycans.

Pathological examination of demyelinated white matter areas has led to the classification of three distinct types of demyelinated lesions: active, mixed active/inactive and inactive lesions ([35,37], Figure 1). Active lesions are highly associated with inflammation and therefore generally contain a relatively high number of inflammatory cells, including resident microglia and infiltrating peripheral monocytes, T cells, and CD20-positive B cells [28,36,38]. Human leukocyte antigen–DR isotype (HLA-DR)-positive macrophages are evenly distributed throughout the demyelinated area [35,37] and based on double expression of IBA1 and microglia marker TMEM119, active lesions consists of approximately 45% microglia-derived macrophages and 55% peripheral-infiltrated macrophages [39]. In addition, infiltrating T cells and CD8-positive tissue-resident memory T cells, which are normally located at the perivascular space, invade the parenchyma of active MS lesions [40]. Strikingly, axonal density is drastically reduced by 30% compared to surrounding non-demyelinated normal-appearing white matter (NAWM) [41]. T cells and macrophages, either microglia or monocyte-derived cells, but also B cells, contribute to demyelination and axonal damage [41,42,43]. Both OPCs and mature oligodendrocytes are present, although at reduced levels compared to NAWM [11,12,13,14]. Oligodendrocyte loss correlates with macrophage infiltration, but not with the amount of T cells, plasma cells and the extent of axonal preservation [11]. Astrocytes acquire a hypertrophic morphology in active MS lesions, i.e., swollen cytoplasm in the cell body and processes, and a reduced process density [37], with the appearance of a neurotoxic phenotype [44].

Inactive lesions are completely demyelinated and are characterised by a sharp lesion border and reduced inflammatory activity. HLA-DR-positive microglia/macrophages are hardly observed in inactive MS lesions, and the presence of T cells is restricted to the perivascular space and comparable to NAWM [28,36,40], while B cells are hardly present [38]. Astrocytes are prominently present in inactive lesions, and have a small cell body and long thin processes that are aligned and form a glial scar [45]. The number of mature oligodendrocytes is low [11], while OPCs are present in about 70% of the lesions [11,13,41,46,47,48]. Within inactive demyelinated lesions, the axonal density is on average 60–70% reduced compared with that in normal tissue of the same area [41,46].

Mixed active/inactive lesions generally display a phenotype between active and inactive lesions regarding the cellular content. Similar to active lesions, the parenchyma is enriched with CD4- and CD8-positive T cells [40], while one-third of mixed active/inactive lesions contain CD20-positive B cells [38]. Mixed active/inactive lesions show a rim of macrophages, and these rims are considered as demyelinating areas [45]. Within the rim, more acutely damaged axons are detected with low numbers of T and B cells [38,40]. Remarkably, macrophages at the rim are mainly microglia-derived, and primarily a pro-inflammatory rather than a pro-regenerative phenotype [12,49]. The latter phenotype is predominantly observed in a perivascular localisation. As in inactive lesions, mature oligodendrocytes are absent in the lesion core [12], while in most lesions, OPCs are present [11,12,13,14]. Astrocytes show varying degrees of morphology and reactivity with hypertrophic astrocytes at the rim, and the formation of an astroglial scar in the core [45].

Remyelinated lesions, or so-called shadow plaques, display no inflammatory activity, i.e., HLA-DR-positive microglia/macrophages and scar-forming astrocytes are absent, although resident B and T cells have been detected [38,40]. The axonal density and concentration of oligodendrocytes generally recover to values close to unaffected tissues [41]. Recent studies revealed that remyelination in shadow plaques proceeds via surviving pre-existing oligodendrocytes [15,16], indicating that demyelination and loss of oligodendrocytes may have been less severe in these areas. Notably, within active and inactive lesions, a variable degree of remyelinated areas are observed, while most mixed active/inactive lesions lack signs of remyelination [45].

The distinct lesion types are observed in all forms of MS, and the frequency of their occurrence is related to disease course and severity. For example, active and remyelinated lesions are most prominent in RRMS [35,36], and mixed active/inactive and inactive lesions are predominant in progressive MS, while the proportion of mixed active/inactive lesions correlates with disease severity [35]. In addition, disease progression correlates with a lower proportion of remyelinated lesions [35]. Remarkably, the presence of B cells is inversely correlated with disease severity [38]. Monitoring the presence of specific types of lesions could be a prognostic tool to predict disease severity and progression. However, for therapeutic decision making, a more accurate characterisation of MS lesion content may be required. Given that gene expression variability is driven by patient-specific effects [50] and that distinct peripheral immune and glial cells are present in MS lesions, different environmental signalling cues in the lesions represent remyelination potential.

### 2.3. Distinct Extracellular Matrix Hallmarks of Demyelinated White Matter MS Lesions

OPC-based remyelination is more efficient than remyelination by surviving mature oligodendrocytes [16,17] and is a natural response to demyelinating injury [10] in experimental models. A non-permissive microenvironment that impairs either OPC recruitment or differentiation results in remyelination failure, leading to chronic demyelination. Several ECM proteins are prominent contributors to the prevention of OPC-based myelin regeneration. In experimental models, the transient deposition of ECM proteins, such as chondroitin sulphate proteoglycans (CSPGs), fibronectin, vitronectin, osteopontin and tenascins-C and R, is an immediate response of astrocytes to demyelination [20,21,51,52,53,54,55,56,57]. Remodelling of the ECM after demyelination is important for early remyelination processes, such as promoting OPC proliferation and migration to the lesioned area and preventing premature OPC differentiation. MS lesions are a result of an incomplete or ineffective remyelination process following acute demyelination. In contrast to experimental models, tenascins-C and -R are decreased in active and mixed active/inactive lesions, most pronounced at the rim (Figure 1) [58]. Also, fibronectin, fibulin-2 and osteopontin, present at very low levels in the parenchyma of the adult CNS, are consistently present in active, inactive and mixed active/inactive lesions [56,59,60,61,62]. Remarkably, CSPGs, such as versican, are increased at the rim of active and mixed active/inactive lesions, while being decreased in the core of active, inactive and mixed active/inactive lesions [63,64,65]. Hyaluronan (HA), a major interstitial ECM constituent of the adult brain, accumulates in the three types of demyelinated white matter MS lesions as high molecular weight hyaluronan (HMW-HA) [66].

In experimental models, the transient deposition of ECM molecules is tightly regulated by MMPs. A major cellular source of MMPs is resident microglia and peripheral-derived macrophages. MMPs, including MMP2, MMP3, MMP7, MMP9, MMP12 and MMP19, are present in active lesions and the rim of mixed active/inactive lesions, while being almost absent in inactive lesions and the core of mixed active/inactive lesions [20,22,67,68,69,70,71,72,73]. The conversion of transient expression to persistent expression makes ECM proteins prone to modification, as exemplified by inflammation-mediated formation of fibronectin aggregates [56,74]. Hence, while the ECM composition in MS lesions changes as a natural response to demyelination, the synthesis and/or clearance of ECM proteins by MMPs is likely disturbed by chronic inflammation, leading to prolonged presence, increased synthesis and/or modification of several ECM proteins in a lesion-specific manner (Figure 1). This not only interferes with OPC differentiation but also retains microglia in a proinflammatory activation state [21,62,65,75,76,77], thereby hindering the resolution of lesions. Evidently, the cellular and ECM composition of white matter MS lesions may determine whether a therapeutic strategy overcomes remyelination failure, indicating that treatment efficacy may differ between the different lesion types.

### 2.4. Neurodegenerative Processes in MS Lesions

**Figure 2:**
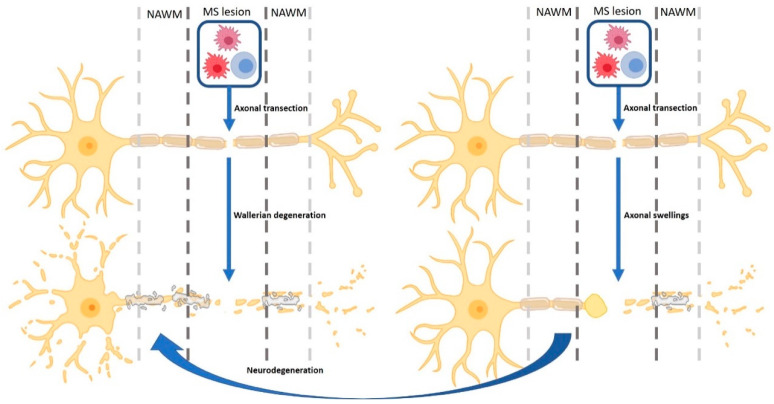
Axonal transection processes are due to both adaptive and innate inflammatory cells. Microglia (red cell), macrophages (pink cell), and T cells (blue cell) cause axonal transection. Axonal transection first manifests as terminal swelling or ovoids as a result of a block in transport of axonal organelles and proteins (**right panel**) and ultimately leads to neurodegeneration through Wallerian degeneration (**left panel**). When no axonal regeneration occurs, the neuron eventually degenerates. MS—multiple sclerosis; NAWM—normal-appearing white matter. The figure is created using Biorender.

**Figure 3:**
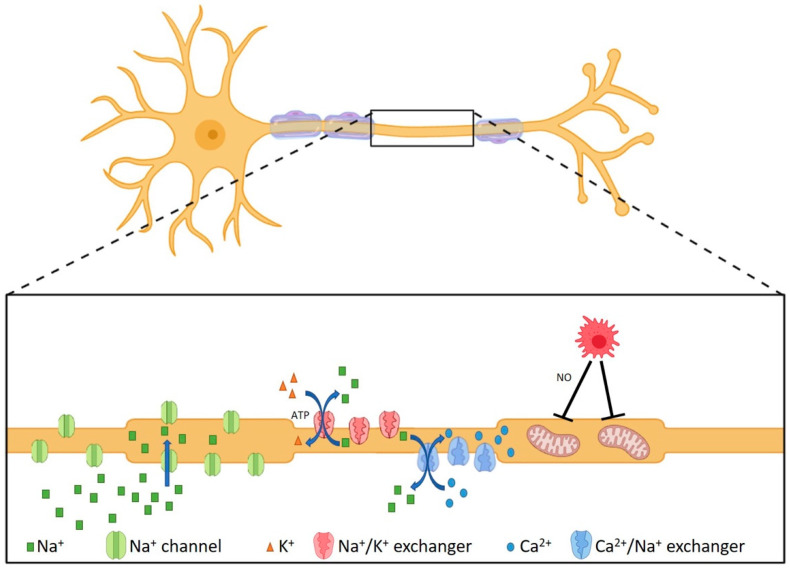
Axonal deterioration of demyelinated axons related to ATP shortage. Demyelination upregulates Na^+^ channels to maintain action potential propagation and therefore results in an increase in intracellular Na^+^. Subsequently, the ATP-dependent Na^+^/K^+^ exchanger restores axonal Na^+^ levels, which requires ATP. However, the ATP availability is insufficient due to the absence of ATP support from oligodendrocytes via myelin. This leads to reversal of the Na^+^/Ca^2+^ exchanger, causing an efflux of Na^+^ and an influx of Ca^2+^. The increased axonal Ca^2+^ levels subsequently activate Ca^2+^-dependent proteases, eventually resulting in axonal degeneration. These processes are further enhanced by nitric oxide (NO) released by macrophages and microglia (red cell), which inhibits axonal mitochondrial ATP production. The figure is created using Biorender.

Axonal injury likely starts at the pre-symptomatic stage of MS, and permanent neurological disability develops when a threshold of axon loss is reached and the CNS compensatory resources are exhausted [78,79]. This hypothesis is supported by the correlation between axonal damage and the expanded disability status scale (EDSS) score, a scale to assess the severity of MS symptoms, and is thought to describe the transition to a progressive disease course [78,79]. Axonal and neuronal degeneration in MS can be either a direct result of inflammation (Figure 2), which causes axonal transection, or a secondary effect of remyelination failure (Figure 3). Persistent demyelination results in diminished metabolic and trophic support from oligodendrocytes [80,81,82,83,84], and increased demands of neuronal ATP levels to maintain axonal function [79,85].

Axonal transection is the main cause of initial moderate disability in most MS patients [78,86]. Axonal transection can be initiated by cytotoxic CD8-positive T cells, but is more commonly induced by activated macrophages and microglia [78,79,87]. Axonal transections manifest as terminal ovoids or swellings, which are present in high density in both active and mixed active/inactive lesions [41,46,78,87,88]. The swellings are the result of impaired transport of axonal organelles and proteins, and are characterised by the accumulation of anterogradely transported proteins, such as amyloid precursor protein. Focal axonal transection may ultimately lead to neurodegeneration [46]. Most axonal transections occur during the process of active demyelination and lead to widespread Wallerian degeneration [41,46,79,89]. Wallerian degeneration describes a phenomenon in which focal axonal damage leads to distal axonal starvation, and therefore explains the underlying diffuse clinical abnormalities and axonal loss in the surrounding white matter [41,46]. When axonal and/or myelin regeneration fails, Wallerian degeneration may lead to further anterograde and retrograde axonal degeneration [87,89,90]. Therefore, current therapies that aim to reduce inflammation limit the number of both direct and indirect axonal transections, and therefore (temporarily) reduce Wallerian degeneration.

Demyelination impairs the efficiency of action potential propagation, leading to redistribution and upregulation of Na^+^ channels along the axon [41,79,85,91]. The increased number of Na^+^ channels along the demyelinated axon requires a large quantity of Na^+^ ions to be pumped back into the extracellular space by the energy-dependent Na^+^/K^+^-ATPase. The demand for ATP exceeds the production capabilities of existing mitochondria, and the Na^+^/K^+^ ATPase pumps, crucial to maintaining ionic gradients, begin to fail [41,79,85,91]. An excess of Na^+^ ions accumulates intracellularly and eventually reverses the Na^+^/Ca^2+^ exchanger that normally transports Na^+^ into the cell and Ca^2+^ out of the cell (Figure 3). The axonal Ca^2+^ influx activates Ca^2+^-dependent proteases, which degrade the axonal cytoskeleton, leading to neurodegeneration [41,79,85,91]. Activated microglia and macrophages produce nitric oxide, which reduces mitochondrial ATP production, and thus enhances the above cascade of neurodegeneration due to demyelination [85]. The anterograde and retrograde degeneration caused by Wallerian degeneration and neurodegeneration as a result of increased energy demands are more prominent in progressive MS, and are observed in inactive lesions [41,85]. Remyelinated lesions lack these neurodegenerative processes [41]. Hence, to prevent neurodegeneration, therapeutic strategies currently focus on promoting remyelination by endogenous OPCs (reviewed in [92,93]) or by exogenous cells (reviewed in [94]).

## 3. MS Prognosis and Treatment Options

**Figure 4:**
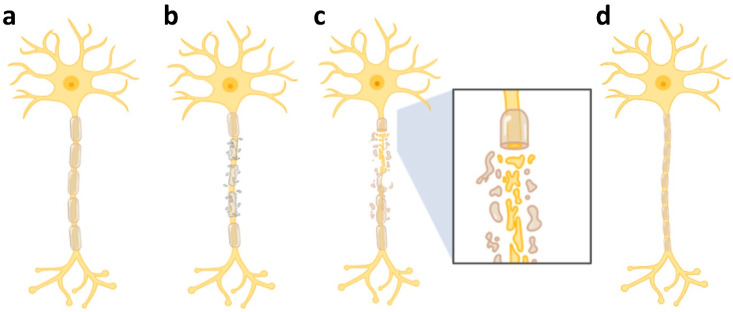
Lesion characterisation based on myelin density and axonal integrity. From left to right: (**a**) normal myelination and normal axon, (**b**) partial demyelinated but otherwise intact axon, (**c**) demyelinated and degenerated axon, and (**d**) remyelinated intact axon. A neuroprotective therapy, such as enhancing remyelination, should be considered when the majority of axons in a demyelinated area are still intact (**b**), while an axon regenerative approach is required for lesions with prominent axonal degeneration (**c**). The figure is created using Biorender.

Knowledge of the cellular and molecular composition of MS lesions is essential for determining therapeutic strategies. For prognosis, i.e., to predict whether MS lesions will or can be repaired, four main aspects of the lesion may be considered for in vivo molecular imaging: (1) inflammatory activity status, (2) myelin density, (3) axonal integrity, and (4) the presence of OPCs. The inflammatory activity status of white matter lesions is highly associated with activation of macrophages and microglia (Figure 1). This histopathological classification corresponds with the MRI classifications regarding active and inactive lesions [7,35,37,95]. On MRI scans, active lesions are marked by T_1_w gadolinium enhancement [7,27]. A subset of mixed active/inactive lesions can be identified by visualisation of a paramagnetic lesion rim, and is associated with a poor prognosis in MS patients [96,97,98]. The paramagnetic rim can be visualised with high-resolution phase images on 1.5, 3, and 7 Tesla MRI systems [99,100,101]. However, phase images can only be acquired with T_2_w* or SWI sequences, which will most likely be included in the new recommended guidelines [95,102]. As MS is a demyelinating disease, the inflammatory activity status does not fully capture disease severity. Therefore, it is logical to also make a distinction between lesions based on myelin density: remyelinated, partial remyelinated/partial demyelinated, and demyelinated (Figure 4). Imaging of myelin with MRI is under development, but histological correlations indicate the need for further refinement [103,104]. Assessment of the myelin density in lesions will not only add to a more accurate lesion characterisation, but monitoring myelin density in lesions is also important for disease prognosis and determination of the eligibility for and efficacy of remyelination strategies. In addition, knowledge of the integrity of axons is essential, i.e., whether axons in the lesions are intact, damaged, or degenerated. MRI methods for axonal integrity exist, but remain poorly validated [105,106]. When most axons are degenerated, treatment should first be directed at axonal regeneration, followed by a (re)myelination strategy. A neuroprotective treatment, such as remyelination, increases axonal survival, restores saltatory nerve impulse conduction, and therefore reduces disease progression and resolves functional deficits. Other neuroprotective non-remyelination-based therapies, such as the application of Na^+^ channel blockers, inhibition of the Na^+^/Ca^2+^ exchanger, or inhibition of Ca^2+^-dependent proteases, may be considered [41,107].

Finally, as most efficient remyelination by endogenous cells is achieved by OPCs [10,16,17], knowledge of the availability of OPCs is desirable. Immunohistochemical analysis on post-mortem brain tissue of MS patients revealed that OPCs are present in approximately 70% of lesions [11,13,41,46,47,48], which requires a different remyelination strategy than when OPCs are absent. When OPCs are present, their differentiation has to be stimulated, but when OPCs are absent, OPCs first have to be activated and recruited to the lesion. Strategies to promote OPC differentiation can be categorised in approaches that directly promote OPC differentiation and approaches that aim to counteract the inhibitory environmental cues that prevent OPC differentiation. Environmental cues, such as the absence of pro-regenerative laminin-2, or the presence of remyelination-impairing fibronectin aggregates, HMW-HA, CSPGs and fibulin-2 have been associated with the lack of remyelination of MS lesions [20,21,62,65,108]. Laminin-2 promotes OPC maturation and survival of oligodendrocytes [109,110,111,112,113], whereas fibronectin, HMW-HA (digestion products), CSPGs, and fibulin-2 inhibit OPC differentiation and myelination [51,56,62,65,66,114,115,116,117,118,119,120,121]. Stimulation of laminin-2 production and/or inhibition of fibronectin, HA and CSPGs may provide potential treatment options. To this end, different ECM-targeting approaches have been developed, including inhibition of CSPG synthesis [53,122,123,124], stimulation of fibronectin aggregate and CSPG clearance [51,67,114,115], prevention of HA degradation [125], and interference with CSPG- and fibronectin-mediated signalling [126,127].

## 4. PET Imaging as a Tool to Stratify Distinct Lesions in MS Patients

Due to its sensitivity and quantitative nature, PET may be used for assessing the biological constituents of MS lesions, provided that suitable tracers for the biological targets of interest are available. Usually, small lipophilic molecules labelled with a radio-isotope are used to assess biological processes in the brain with PET imaging. The characteristics of the labelled molecule determine which biological process is visualised. For instance, [^18^F]FDG (radio-labelled glucose) is used to assess cerebral glucose metabolism, and [^11^C]PiB to determine the presence of amyloid-beta deposits, a pathological marker of Alzheimer’s disease. Several comprehensive reviews discussed the use of PET-imaging in MS patients, but focus mainly detection of neuroinflammation and/or discussed emerging PET tracers for imaging myelin, microglia activation, and reactive astrocytes [128,129,130,131,132,133,134,135]. In contrast to these excellent overviews, in the following, we will also discuss how PET imaging may allow visualisation and quantification of the dynamic cellular and molecular alterations of the four determinants for white matter MS lesions. We will address current available PET tracers and provide an overview of interesting biological aspects that can serve as potential targets for PET tracers of OPC availability and the molecular ECM environment. The latter will not only aid in lesion stratification, but also in the prognosis of lesion progression and therapy decision making of remyelination-based therapies.

### 4.1. PET Tracers for Cellular and Molecular Characteristics of MS Lesions

#### 4.1.1. Inflammatory Activity Status

Quantitative information about the inflammatory activity within the lesion can be obtained by PET imaging (Table 1). Traditionally, PET tracers that target the overexpression of TSPO in activated microglia are used for this purpose. Using PET imaging of TSPO, the extent of inflammation was found to correlate with clinical disability [136,137,138,139], and longitudinal changes in neuroinflammation upon treatment could be detected [140]. However, identification of the type of immune cells involved (e.g., microglia, macrophages, astrocytes, B cells, T cells) is not possible with MRI or PET imaging of TSPO. As TSPO is upregulated in activated microglia and macrophages, and to some extent in reactive astrocytes and vascular endothelial cells, TSPO tracers cannot distinguish between these innate inflammatory cells or their activation phenotype [141,142]. Recently, [^11^C]SMW139 PET has been investigated with the aim of visualising P2X7 receptor expression on pro-inflammatory microglia and macrophages in MS patients [143]. However, although P2X7 receptors are expressed by pro-inflammatory microglia, they are also expressed by anti-inflammatory microglia, and to a lesser extent by astrocytes, oligodendrocytes, and neurons [144,145]. Therefore, PET tracers to visualise P2X7 receptors cannot fully discriminate between cell types. In addition, the adenosine 2a receptor (A2AR) as a marker for reactive astrocytes has been imaged with [^11^C]TMSX PET in MS, Parkinson’s disease, and Alzheimer’s disease [146,147,148]. However, the A2AR is expressed in many cells, such as T cells, macrophages, microglia, monocytes, NK cells, endothelial cells, and neurons [149,150,151,152]. Hence, more specific targets are required. Several targets for PET tracers are cell type-specific. For example, monoamine oxidase-B (MAO-B) is primarily found in activated astrocytes and, to a much lower extent, in neurons. MAO-B can be visualised with the PET tracer [^11^C]DED [153,154], which so far has been primarily used to detect glial scars in Alzheimer’s disease patients. Other MAO-B tracers are currently still in a developmental phase and have only been assessed with autoradiography on post-mortem tissue [155]. The colony-stimulating factor-1 receptor (CSF1R), on the other hand, is almost exclusively expressed by microglia, irrespective of their phenotype. Recently, PET tracers, such as [^11^C]CPPC and [^11^C]GW2580, are developed for this target [156,157]. However, [^11^C]CPPC proved to be a substrate of the P-glycoprotein efflux pump in the BBB and therefore less suited for PET imaging [158]. Cell type-selective PET tracers, such as [^11^C]DED and [^11^C]GW2580, could enable better characterisation of the innate inflammatory response. In addition to the aforementioned tracers, more tracers for neuroinflammation have been developed (see Table 1) and are thoroughly discussed in other recent reviews [128,159,160].

So far, attempts to image adaptive inflammatory cells in the brain have largely remained futile. In humans, radio-labelled anti-CD20 antibodies, which are specific for B cells, but not plasma cells [196], and radio-labelled anti-T cell antibodies, such as anti-CD3, anti-CD4, or anti-CD8 [197,198,199], as well [^18^F]FB-IL2, for T cell imaging have been employed, but none of these protein-based PET tracers are able to cross the BBB [200].

Taken together, despite significant efforts to develop specific PET tracers for the visualisation and quantification of distinct inflammatory cell populations, this remains a challenging endeavour. Although TSPO PET imaging has been widely used, the elucidation of TSPO expression through a wide cellular spectrum underscored the necessity for more selective tracers. For the innate immune system, [^11^C]GW2580 has shown promise for imaging microglia, while [^11^C]acetate and [^11^C]DED have demonstrated potential for astrocyte visualisation. However, the development of PET tracers targeting cells of the adaptive immune system continues to pose significant challenges.

#### 4.1.2. Myelin Density

Current PET tracers for myelin imaging can be divided into two main categories: PET tracers developed for amyloid imaging and PET tracers specifically developed for myelin imaging (Table 1). Amyloid PET tracers bind to beta-sheets in beta-amyloid deposits, but also to beta-sheets in myelin basic protein (MBP), a major protein constituent of myelin. Consequently, amyloid PET tracers, such as [^11^C]PiB, have been repurposed for myelin imaging in MS patients [174,175,176,177,179]. These studies consistently reveal a lower tracer uptake in MS lesions as compared to NAWM [174,175,176,177,179]. In addition, longitudinal studies with repurposed amyloid PET tracers indicate that a decrease in myelin density corresponds with higher clinical disability [175,178,201,202]. PET tracers specifically developed for myelin imaging, such as [^11^C]MeDAS, also bind to the beta-sheets of MBP [180,181], and differentiate myelin densities across MS lesions [104,180,185,186]. The advantage of tracers such as [^11^C]MeDAS is that they do not accumulate in amyloid deposits.

A general drawback of myelin imaging with PET is that a decrease in or lack of myelin in MS lesions results in a lower signal than the surrounding tissue, a so-called coldspot. Detection of coldspots is challenging due to partial volume effects that cause spill-over of signal from surrounding intact myelin to the MS lesion, which results in an overestimation of the myelin density in the lesion. An alternative approach to assess myelin damage may be the visualisation of myelin debris, which causes hotspots that are generally easier to detect. A potential strategy for in vivo molecular imaging of myelin debris is the application of a radio-labelled tracer against damaged MBP (dMBP) of which the epitope on MBP is only visible upon demyelination [9,203,204,205]. However, this relies on the tracer being able to cross the BBB and on myelin debris still being present in MS lesions. A potential confounding factor could be the signal derived from dMBP, which is already phagocytosed by microglia and macrophages. Potentially, myelin damage may also be visualised by axonal potassium channels. Upon demyelination, axonal potassium channels that are normally covered by myelin become exposed and increase in expression. Imaging of these specific axonal potassium channels using [^18^F]3F4AP seems promising, albeit not yet used in humans [187,206].

It is important to emphasise that myelin imaging may not reflect axonal integrity. Axons degenerate rapidly distal to the site of a transection caused by inflammation, whereas myelin can persist for a long time after proximal fibre transection. Such remaining myelin sheaths will form empty tubes, or degenerating ovoids [78]. Therefore, imaging of myelin should not be used for assessing axonal integrity, as it may lead to “false positive” results. Furthermore, myelin damage does not necessarily lead to immediate axonal degeneration, hence, upon myelin damage, myelin restoration therapies can promote axonal survival.

#### 4.1.3. Axonal Integrity

[^18^F]FDG is classically used as a marker for neuronal integrity under the assumption that the majority of glucose-consuming cells within the CNS are neurons [207]. However, [^18^F]FDG uptake does not always correctly reflect neuronal integrity, because glucose consumption by neurons is highly dependent on brain activity and inflammatory cells are also major glucose consumers [208,209]. Since inflammatory cells can be present in (active) MS lesions, [^18^F]FDG is less suitable as a PET tracer to assess axonal integrity in (active) MS lesions. Another approach to image axonal integrity might be visualisation of tau protein. Tau stabilises axonal microtubules, which become soluble upon axonal damage [195]. Tau has been detected in the CSF of MS patients [210], indicating that tau may be a marker for neurodegeneration in MS. PET imaging of tau to assess neurodegeneration has already been employed in other neurodegenerative diseases, including Alzheimer’s disease, progressive supranuclear palsy, corticobasal syndrome, Down’s syndrome, Parkinson’s disease, and dementia with Lewy bodies [195]. Neuronal signal transmission occurs via synapses, which have a pre-synaptic and post-synaptic part. Upon neurodegeneration, the number of synapses decreases due to loss of pre-synaptic or post-synaptic neurons. Presynaptic synaptic loss may be measured with [^11^C]UCB-J, a tracer for presynaptic vesicles, which might therefore be a marker of neuronal integrity [211,212]. However, this tracer has not been used in MS patients yet, and as [^11^C]UCB-J PET measures synaptic density, it will likely depict primarily neuronal integrity in grey matter and not axonal integrity in white matter.

Therefore, given the limitations of [^18^F]FDG in accurately reflecting neuronal integrity in active MS lesions due to the contribution of inflammatory cell metabolism, alternative neurodegeneration markers, such as tau PET imaging, hold potential for evaluating neuronal integrity in MS lesions, though their applicability in MS has yet to be established.

#### 4.1.4. Oligodendrocyte Progenitor Cells

Currently, there are no PET tracers that can be used for the detection of OPCs. A potential biological target for imaging of OPC availability might be platelet-derived growth factor receptor alpha (PDGRFα) [213]. For example, anti-PDGRFα antibodies could be radio-labelled for PET imaging; however, due to the poor BBB penetration of antibodies, such a tracer might only be suitable for active lesions. Small-molecule ligands, such as PDGRFα inhibitors (e.g., AG1296 [214]), may be suitable for all types of lesions, provided they are sufficiently lipophilic to cross the BBB. During early OPC differentiation, which highlights the onset of remyelination, G protein-coupled receptor 17 (GPR17) and breast carcinoma amplified sequence 1 (BCAS1) are transiently upregulated in white matter OPCs [215,216]. Although their application in PET imaging has not yet been established, GPR17 and BCAS1 could be interesting targets to determine in an early phase the efficacy of remyelination therapies. Since OPCs are distributed throughout the brain [217], accurate delineation of MS lesions will be important. Since PET lacks structural information, usually a T_1_w MRI is acquired and co-registered with the PET images for accurate analysis. By applying additional T_2_w and T_2_w-FLAIR MRI, accurate lesion delineation can be performed for OPC imaging. Hence, small-molecular lipophilic PDGFRα inhibitors likely represent the most promising target for PET tracer development to assess OPC availability, necessary for determining whether OPCs need to be attracted (in case of absence) or stimulated (in case of presence) to promote myelin regeneration in MS lesions.

#### 4.1.5. Extracellular Matrix

Several PET tracers have already been employed for assessing ECM in other diseases. For instance, ^18^F-labeled and ^64^Cu-labeled tenascin-C aptamers and several radio-labelled antibodies against cellular fibronectin have been evaluated in experimental models of cancer [218,219,220]. Studies investigating collagen with the tracer ^64^Cu-CBP7 for pulmonary fibrosis have also been performed [221]. In addition, currently available ligands, such as antibodies or large proteins, for laminin-2, fibronectin, CSPGs and fibulin-2 could be of interest. However, these PET tracers are all relatively large and likely will not cross the BBB. Thus, small-molecule ligands for these ECM targets that can be radio-labelled for PET imaging are still awaited.

### 4.2. Potential Avenues for Novel PET Tracers for Brain Imaging: Targeted Blood–Brain Barrier Crossing

An important limitation for many potential PET tracers for MS lesion characterisation is the transport of tracers across the BBB. To passively cross the BBB, a PET tracer should be a small molecule (<400 D) with a relatively high lipophilicity (logP > 2) [23,24,222]. As mentioned in previous sections, several antibody- and peptide-derived PET tracers for immune cells and ECM proteins already exist, but have proved unable to cross the BBB. Building on neuroradiological developments in neuro-oncology, a strategy for small (peptide-derived) PET tracers could involve their encapsulation in self-assembling biodegradable nanocarriers coated with hyaluronic acid (HA) [223]. These HA-coated nanocarriers are capable of crossing the BBB primarily through receptor-mediated transcytosis via its receptor CD44, whose expression on brain endothelial cells is significantly upregulated under inflammatory conditions. However, when performing PET imaging with nanocarriers, it is unclear whether the PET image reflects the transcytosis or the biological target of interest. Most likely, the image would reflect both, making such a method less suitable for PET imaging of MS lesions.

**Figure 5:**
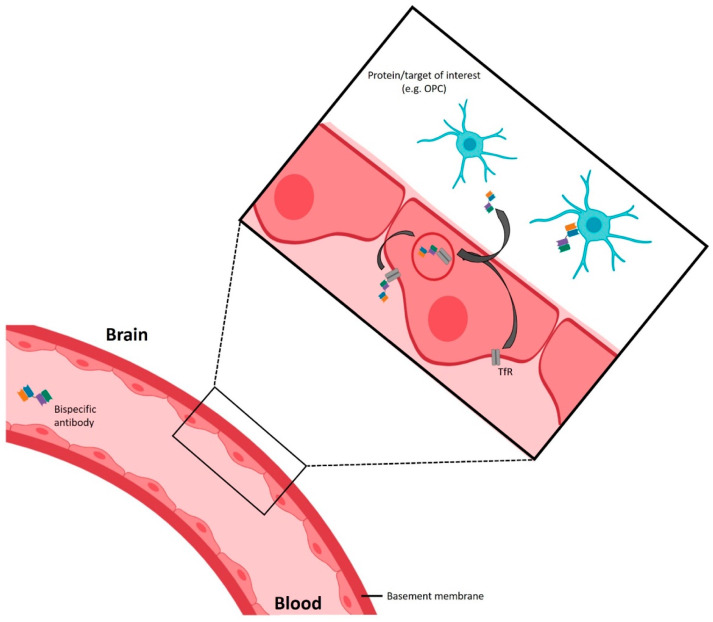
Transport of radio-labelled bispecific antibodies across the blood–brain barrier (BBB). Radio-labelled bispecific antibodies directed against e.g., the transferrin receptor (TfR) and the protein/target of interest bind to TfR at the luminal ‘blood’ side of the BBB. Following receptor-mediated transcytosis, the radio-labelled bispecific antibodies are released at the abluminal ‘brain’ side of the BBB, enabling their binding to the protein or biological phenomenon of interest and visualisation by PET imaging. The figure is created using Biorender.

Radiolabelled antibodies and related proteins are generally large molecules with a low lipophilicity and thus can only be used to image biological processes in the brain when the BBB is compromised, as in active MS lesions. A recent development might extend the use of radio-labelled antibodies to the brain [224,225,226,227]. After modification of the antibody with a ‘transport sequence’ that binds to specific receptors at the luminal ‘blood’ side of the BBB, brain accumulation of antibodies could be achieved by receptor-mediated transcytosis (Figure 5). More specifically, binding of these antibodies to the ‘transport receptor’ induces endocytosis of the antibody–receptor complex, followed by exocytosis at the abluminal ‘brain’ side of the BBB. Potential receptors that might be suitable for this transcytosis strategy are the insulin receptor, LDL-related protein type 1 receptor, and transferrin receptor 1 (TfR1) [228,229]. Preclinical studies in experimental models of Alzheimer’s disease successfully employed this kind of strategy. A radio-labelled bispecific antibody that targets both the transferrin receptor 1 at the BBB and soluble amyloid-beta protofibrils was used [224,225,226]. Likewise, radio-labelling of the antibody–transporter conjugates (e.g., anti-dMBP or anti-PDGRFα conjugated with transferrin) could be applied as PET tracers that can detect the target or cell type of interest in MS lesions with a similar accuracy as immunohistochemical methods. However, the interpretation of the images could be complicated, since in this approach the PET signal not only depends on the concentration of the biological target but also on the activity of the BBB transporter.

## 5. Considerations for PET Imaging of MS Lesion Characteristics in Clinical Practice

The most important biological processes in MS lesions that might be characterised with imaging are inflammation, myelin density, axonal integrity, and the presence of OPCs. Whereas ECM constituents also have an important effect on the efficacy of de- and remyelination, assessment of these targets is primarily of interest for therapeutic purposes. Given the interindividual heterogeneity in inflammatory activity, axonal integrity, OPC availability, and remyelination capacity, personalised therapeutic approaches may be required to stop disease progression. In fact, the complexity and regional diversity of MS pathology have not only challenged the development of therapeutic strategies for progressive MS, but also limited the successful translation of candidate regenerative drugs to the clinic [230,231,232,233,234].

Current clinical MRI protocols, although important in diagnosing MS and providing superior anatomical images, do not allow for a reliable and quantitative analysis of the distinct classified MS lesion types. By applying PET imaging, prior knowledge of the type of lesions, axonal integrity, OPC availability or molecular environment may be obtained, which will aid the stratification of patients eligible for participation in clinical trials, enabling the inclusion of only those MS patients that are predicted to be responsive to the candidate therapeutic agent. A hurdle is that radiation exposure is limiting the number of repeated PET scans that can be performed in an individual patient. Due to current technical developments in PET imaging, sensitivity and resolution of PET scans increase, which enables the acquisition of PET scans with a lower tracer dose, and, thus, lower radiation exposure. Therefore, the future clinical application of PET scans will be more easily justified, as the amount of radiation exposure will be reduced more and more.

An additional consideration with PET imaging is the high cost and the limited availability of PET-MRI scans in hospitals. However, PET imaging of specific disease processes could support improved patient selection for expensive treatments and allow more personalised approaches to myelin regenerative therapies. This will ultimately limit (the costs of) accrual of clinical disability, and the cost of the scans can be offset against the savings on medication costs. Introducing and approving new PET tracers into clinical practice also requires time spent performing clinical studies to demonstrate safety and efficacy. These issues have to be addressed before widespread use of PET imaging in MS disease management is feasible.

## 6. Conclusions

The growing understanding of the biological mechanisms underlying MS pathogenesis provides new opportunities for PET tracer development, particularly in the context of drug discovery and therapy evaluation. While PET imaging holds potential for assessing disease prognosis for individual lesions, treatment selection, and disease progression, its clinical utility will only be justified when effective neuroprotective or remyelination therapies become available. Conversely, PET imaging may play a crucial role in drug development by enabling the assessment of inflammatory activity, myelin density, axonal integrity, and OPC availability, which are key factors in evaluating remyelination strategies.

Future applications of combined PET-MRI cameras could facilitate quantitative lesion characterisation and aid in predicting treatment efficacy, ultimately supporting more personalised and evidence-based treatment strategies. Specifically, imaging markers of axonal integrity, myelin density, and OPC dynamics may guide the selection of therapies aiming to stimulate neuronal regeneration, OPC recruitment, or OPC differentiation. Furthermore, PET imaging of remyelination-inhibitory ECM components, such as fibronectin and CSPGs, may enhance our ability to predict treatment response. Recent advances in radio-labelled antibodies, nanomedicine and biomedical engineering hold promise for enhancing tracer delivery to the brain, paving the way for new theranostic applications for PET imaging in MS. However, difficulties with disentangling endocytosis tracer delivery effects and target binding remain an unsolved issue, which currently hampers clinical utilisation of transcytosis-dependent tracer delivery, such as the use of nanoparticles or bispecific antibodies.

## Figures and Tables

**Table 1:** Examples of current available PET tracers that may be employed for the visualisation of biological processes in MS lesions.

Imaging Target	Relevant Process/Cells	Tracer	Status	References
Inflammation				
TSPO	activated microglia/macrophages, astrocytes, endothelial cells	[^11^C]PK11195 [^11^C]PBR28 [^18^F]PBR06 [^18^F]PBR111 [^18^F]GE-180 [^11^C]DPA713 [^18^F]DPA714	clinical research	[161,162,163,164,165,166,167,168,169,170,171]
P2X7 receptor	pro-inflammatory microglia, astrocytes, OPCs	[^11^C]SMW139	clinical research	[143]
CSF1 receptor	activated microglia	[^11^C]GW2580	Experimental models	[156,157]
MCT1	activated astrocytes	[^11^C]acetate	clinical research	[172]
Adenosine 2A receptor	T cells, macrophages, microglia, monocytes, NK cells, endothelial cells, neurons	[^11^C]TMSX	Clinical research	[146,173]
monoamine oxidase-B	activated astrocytes	[^11^C]DED	experimental models	[153]
**Myelin Density**				
amyloid beta & MBP	in MS pathology, assessment of myelin integrity	[^11^C]PiB [^18^F]Florbetaben [^18^F]Florbetapir	clinical research	[174,175,176,177,178,179]
MBP	assessment of myelin integrity	[^11^C]MeDAS	clinical research	[180,181,182,183,184,185,186]
axonal potassium channels	assessment of myelin integrity	[^18^F]3F4AP	Experimental models	[187]
**Axonal Integrity**				
synaptic vesicle protein 2a	synaptic density	[^11^C]UCB-J	clinical research	[188]
glucose consumption	neurodegeneration	[^18^F]FDG	clinical research	[189,190,191,192,193]
tau	neurodegeneration	[^18^F]Flortaucipir [^18^F]MK-6240	clinical research	[194,195]

Abbreviations: CSF1 receptor—colony stimulating factor-1 receptor; MBP—myelin basic protein; MCT—monocarboxylate transporter; MS—multiple sclerosis; OPC—oligodendrocyte progenitor cell; TSPO—18kD translocator protein.

## Abbreviations

BBB—blood brain barrier; CNS—central nervous system; CSF—cerebrospinal fluid; CSPG—chondroitin sulfate proteoglycan; dMBP—damaged myelin basic protein; ECM—extracellular matrix; EDSS—expanded disability status scale; HA—hyaluronan; HMW-HA—high molecular weight hyaluronan; MAO-B—monoamine oxidase-B; MBP—myelin basic protein; MCT—monocarboxylate transporter; MMP—matrix metalloproteinase; MRI—magnetic resonance imaging; MS—multiple sclerosis; NAWM—normal appearing white matter; NO—nitric oxide; OPC—oligodendrocyte progenitor cell; PET—positron emission tomography; PLP—proteolipid protein; PPMS—primary progressive multiple sclerosis; QSM—quantitative susceptibility mapping; RRMS—relapse remitting multiple sclerosis; SPMS—secondary progressive multiple sclerosis; SWI—susceptibility weighted imaging; T_1_w—T_1_ weighted; T_2_w—T_2_ weighted; T_2_w-FLAIR—T_2_ weighted fluid attenuation inversion recovery; TfR—transferrin receptor; TSPO—18kD translocator protein.
